# Foundry: Early learnings from the implementation of an integrated youth service network

**DOI:** 10.1111/eip.13181

**Published:** 2021-05-18

**Authors:** Steve Mathias, Karen Tee, Warren Helfrich, Krista Gerty, Godwin Chan, Skye Pamela Barbic

**Affiliations:** ^1^ Foundry, Providence Health Care Vancouver British Columbia Canada; ^2^ Faculty of Medicine University of British Columbia Vancouver British Columbia Canada; ^3^ Department of Psychiatry University of British Columbia Vancouver British Columbia Canada; ^4^ Department of Psychiatry, Providence Health Care St. Paul's Hospital Vancouver British Columbia Canada; ^5^ Centre for Health Evaluation and Outcome Sciences Vancouver British Columbia Canada; ^6^ Providence Health Care Research Institute Vancouver British Columbia Canada; ^7^ Department of Occupational Science and Occupational Therapy University of British Columbia Vancouver British Columbia Canada

**Keywords:** integrated youth services, mental health, youth

## Abstract

**AIMS:**

To provide the first profile of the demographic and service characteristics of young people (aged 12–24 years) who access Foundry, a provincial network of integrated youth health and social service centres in British Columbia, Canada and to share early learnings about implementation and service innovation.

**METHODS:**

Using a retrospective chart review, we conducted a census of all young people accessing a Foundry centre in a ‘proof of concept’ phase. Six centres were assessed between October 2015 and March 2018. Data included demographics, mental health service access history, service type the youth was seeking, and information about how they found out about the centre.

**RESULTS:**

A total of 4783 young people presented during this proof of concept period, for a total number of 35 791 visits. The most frequently accessed category of service was mental health/substance use (57%) followed by physical health (25%). Young people were most likely to be female, aged 15–19, and White. Youth demographic characteristics showed an over‐representation of Indigenous and LGBTQ2 youth and under‐representation of males and youth aged 20–24. Youth were most likely to learn about Foundry from a friend (44%) or family member (22%). Most youth (58%) reported that they would have gone ‘nowhere’ if not for Foundry.

**CONCLUSIONS:**

Foundry is a model of integrated health and social services delivery, focused on early intervention, prevention and accessibility, driven by the needs and priorities of young people and their families. Leveraging international integrated youth health service evidence, the model addresses urgent priorities in Canadian health service delivery.

## 1 INTRODUCTION

In Canadian and North American samples, mental health and substance use (MHSU) disorders affect 1 in 4 young people aged 12–24 years (Kessler et al., 2005; Statistics Canada, 2011), the highest incidence of mental disorders of any age group (Gore et al., 2011; Statistics Canada, 2015). An estimated 70% of MHSU problems in Canadian young adults have an onset occurring during childhood or adolescence (Addington et al., 2018; Canadian Institutes for Health Information, 2018; Orpana et al., 2018). This represents a population adjusted lifetime economic burden of nearly $200 billion on the Canadian economy (Mental Health Commission of Canada, 2013). One of the most significant challenges in MHSU services is how to engage young people early in their illness trajectory (Kidd et al., 2014; Slade et al., 2014; Williams et al., 2012). Between 2007 and 2017, there was a 66% increase in emergency department visits in Canada, and a 55% increase in hospitalizations of children and youth (age 5–24 years) due to mental health concerns (Canadian Institutes for Health Information, 2018; Lang et al., 2018). While mental health literacy is increasing (Andrade et al., 2014; Jorm, 2000; Krishan et al., 2012), and more young people and their families are seeking help for MHSU problems (Hetrick et al., 2017), there is an absence of accessible and integrated, low‐barrier services, leading to increased utilization of expensive and inappropriate emergency departments by young people and poor social and health outcomes (Barbic et al., 2019; Brimblecombe et al., 2017; Farris et al., 2018; Gmitroski et al., 2018; Hobbs Knutson et al., 2018; MacDonald et al., 2018; Snyder et al., 2017; Tylee et al., 2007; Wissow et al., 2017).

In response, several nations including Australia, France, Ireland, and Canada, and peak bodies within, are re‐thinking the delivery of youth health services, specifically re‐imagining how systems of care can be designed around the needs and priorities of young people and their caregivers (Halsall et al., 2019, 2020; Hetrick et al., 2017). In Australia, a national initiative called *headspace*™ was developed to provide early intervention mental health services to youth, along with an active health promotion infrastructure to support the well‐being of all youth. Other programs around the world include Les Maisons des Adolescents in France, Jigsaw in Ireland, Youth Wellness Hubs Ontario (YWHO) and Foundry, both in Canada (Cross & Hickie, 2017; Hetrick et al., 2017).

Foundry is located in British Columbia (BC), Canada's western most province. BC has a population of 5.5 million, with 2.3 million living in Greater Vancouver, the third‐largest metropolitan area and one of the most ethnically and linguistically diverse cities in Canada (Statistics Canada, 2011). Five health authorities serve the province, providing direct or contracted health services. The province is also supported by a First Nation Health Authority to support the planning, designing, managing, and funding of Indigenous health services. Primary care services are delivered by family doctors or nurse practitioners. Young people between the ages of 12–18 may also receive mental health services from the Ministry of Children and Families. Most social and some health services are delivered by various community not‐for‐profit organizations. Although the system has many working parts, it has been criticized for being difficult to navigate and burdensome for young people and their families.

In 2015, a Working Group was assembled in BC to work closely with young people and families to co‐create a youth and family‐centred model of care to meet their accessibility needs. The co‐design process included experience mapping in diverse BC communities, branding exercises, service prototype creation, and training to actively engage youth from different backgrounds to build a common vision for system change (Hackett et al., 2018; Hawke et al., 2020). Guided by Canada's Mental Health Strategy (The Mental Health Commission of Canada, 2012), the team prioritized engaging young people and their families to describe their goals for health and wellness. Supported by Henderson and colleagues' co‐creation work (Hawke et al., 2020; Heffernan et al., 2017; Henderson et al., 2018), the team hired, trained, and engaged young people and families to inform and support decisions that would impact service provision. Priorities included providing safe, non‐judgmental care, information and resources, and work to reach young people earlier. Other youth priorities included bringing health and social services together in a single, youth‐friendly place to make it easier for young people and families to find the care, connection, and support that they needed.

The process also included engaging international and national partners (i.e., headspace, Jigsaw, YWHO, and ACCESS Open Minds) to share learnings from their organizations to support the development of Foundry's proof of concept and evaluation framework (Hetrick et al., 2017). Some of these members also supported two Strategy for Patient Oriented Research projects to produce a preliminary conceptual and measurement model for understanding the mental health and recovery needs of youth with mental illness (Barbic et al., 2019). The information extracted from this project helped inform the team on how to grow, support, and sustain the capacity for a collaborative, interdisciplinary and innovative patient‐ and family‐oriented care environment.

Foundry's proof of concept phase also included an exhausting branding to create the youth friendly, non‐stigmatizing Foundry brand in 2015. Between 2015 and 2018, ‘Foundry’ rolled out as a network of six integrated youth centres with complementary online tools and resources for youth aged 12–24, with the vision of ‘transforming access to care’ (Mathias, 2018). This was called Phase 1 and is the focus of this paper. As part of a growing international movement (Henderson et al., 2017; Hetrick et al., 2017), the focus of Phase 1 was to develop and implement integrated early intervention and prevention services to support young people by physically bringing health and social services together in one place, while providing a Foundry lead agency funds ($700 k Cdn) with which to buy missing services or resources.

**FIGURE 1:**
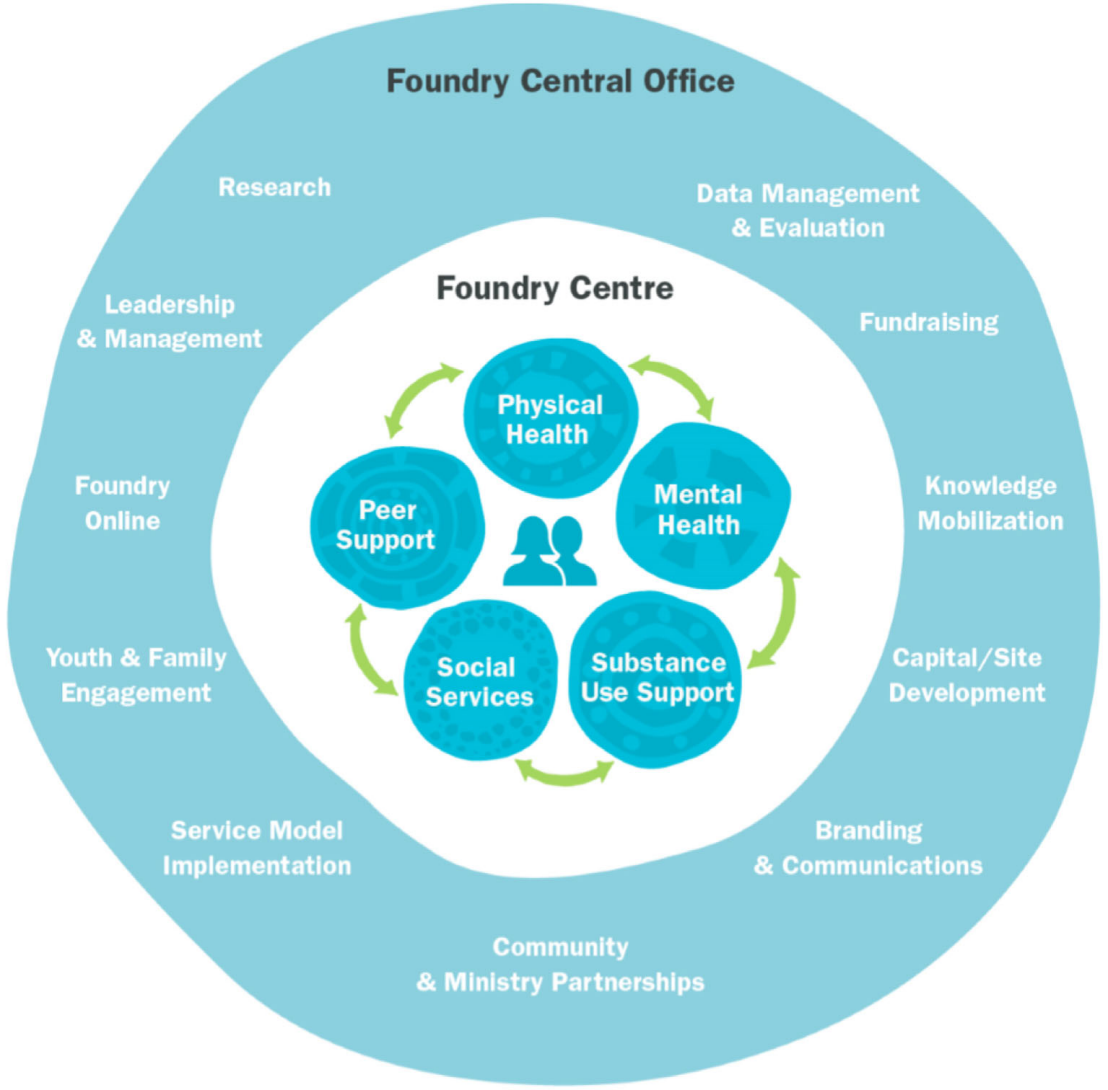
Foundry theory of change. Achieving Foundry's vision—Transforming access to health and social services for youth and their families in BC—Requires the full and meaningful integration of services in each Foundry Centre. Efforts to achieve integration began with the BC Integrated Youth Services Initiative (BCIYSI) proposal and the convening process and carried on through the development of each centre consistent with Foundry's comprehensive service model described above. The end result are services—Both centre‐based and online—That are seamless, relationship‐based, and empower youth and their families to immediately access the support and care they need

Today, Foundry is a partnership between young people, their families, and more than 200 government and non‐profit partners, working together under the Foundry banner in communities across BC. As shown in Figure 1, services at each centre include primary care (physical and sexual health), mental health, substance use, peer support and social services (e.g., employment, housing, and income assistance). Similar to global partner organizations (Hetrick et al., 2017), services are accessed individually or concurrently, and staff and organizations work collaboratively so that young people experience seamless care.

*Phase 1*: Funded by the provincial government and philanthropy, the first centre, Foundry Vancouver‐Granville, was established in 2015 to serve a predominantly inner city population in Vancouver. Replacing the Working Group, a provincial support team (Foundry Central Office or FCO) was established to oversee the complex process of establishing Foundry centres, beginning with the selection of communities via a formal request for proposals. Twenty‐five organizations representing communities from across BC responded. Working with the FCO, five Proof of Concept centres (Abbotsford, Campbell River, Kelowna, North Vancouver, Prince George) were opened in each of the BC health authorities between (1 October 2015–31 March 31 2018). A developmental evaluation of the Foundry implementation process, including the work done by the Foundry Central Office, captured how Foundry impacted the young people and families who accessed services (Salmon et al., 2018; Tee et al., 2018).

The purpose of this paper is to summarize early learnings from the Foundry Proof of Concept Phase. These learnings, combined with our developmental evaluation informed the development of Phases 2 and 3 Foundry Centres (to be reported elsewhere). It is also anticipated that the results from this evaluation will support jurisdictions creating similar models, including provincial leaders in Canada. Internationally, we hope this knowledge contributes to a growing body of new evidence about integrated youth services.

## 2 METHODS

### 2.1 Participants and procedures

Data were collected on all young people accessing services at six operational Foundry centres, during the period of October 2015 to March 2018. All centres reported service count and type data. In four of six centres (excluding Vancouver and Abbotsford), demographic data were collected from each youth at the time of their first visit to a Foundry centre and in each subsequent visit (if they returned). Data were entered into a secure data management platform and aggregated for analysis. Under the Tri‐Council Policy Statement on Ethical Conduct for Research Involving Humans, this evaluation data was distinguished from research requiring research ethics board review as they were collected as part of a quality assurance study of a proof of concept in a ‘real world’ setting.

### 2.2 Measures

The forms captured basic demographic data (age, preferred pronoun, gender, ethnic/cultural background, guardianship, living situation, educational/vocational status, mental health service access history), information about the type of services sought (physical health, sexual health, mental health, substance use, youth peer support or social service), information about how the youth found out about Foundry and where they would have sought services had a Foundry centre not been available to them.

### 2.3 Analysis

We analysed data using R (R Core Team, 2017). We conducted descriptive analyses for all sociodemographic and clinical characteristics using all available data, calculating median and interquartile range for continuous variables and proportions (*n*/*N*, %) for categorical variables.

## 3 RESULTS

Service utilization data were collected from six centres, yielding information about total number of services provided to 4783 unique youth. Total visits recorded for the proof of concept period was 35 791. Descriptive data available from four centres are summarized in Table 1. This represented 40% of the total sample accessing the centres. The 40% reflects concurrent efforts to implement a province‐wide data collection platform with all staff and clinicians during this phase.

**TABLE 1:** Participant demographics (*n* = 1886)

Variable	
Age	
Mean (SD)	17.7 (3.0)
Median	18
Range	12–25
Age breakdown (*n*, %)	
12–14	323 (17)
15–19	1185 (63)
20–24	378 (20)
Gender (*n*, %)	
Male	599 (36)
Female	939 (55)
Non‐binary	149 (9)
Education (*n*, %)	
Less than high school	105 (6)
High school	819 (50)
Training school	16 (1)
College	95 (6)
University	170 (10)
Not in work/study	374 (23)
Other/unspecified	60 (4)
Ethnicity (*n*, %)	
White	1247 (66)
Chinese	43 (2)
Filipino	36 (2)
Other	560 (30)
Sexual orientation (*n*, %)	
Heterosexual	1111 (70)
Bisexual	223 (14)
Gay and lesbian	56 (4)
Pan sexual	47 (3)
Asexual	8 (<1)
Questioning	22 (<1)
Other	125 (8)

### 3.1 Social determinants of health

#### 3.1.1 Age and gender

As shown in Table 1, 76% of youth accessing services were aged 19 years or younger (*M* = 17.7, SD = 3.0). The age and gender distribution of youth accessing services showed a similar pattern for male and female clients aged 12–14 years and 20–24 years. A relatively higher proportion of female youth accessed services in the 15–19 age range (from 3% to 5% more); however, this difference was accounted for by higher utilization of sexual health services by females in this age range.

#### 3.1.2 Ethnicity

Two‐thirds (66%) of young people self‐identified as White, followed by young people that identified as indigenous (14%). As shown in Table 1, South Asian and Chinese youth were notably under‐represented as per provincial norms.

#### 3.1.3 Sexual orientation

One in three (30%) youth accessing Foundry services self‐identified as having a sexual orientation other than heterosexual. Data from the Canadian Community Health Survey (Statistics Canada, 2012) found that just 1.7% of Canadians 18 years and over identified as gay or lesbian and just 1.3% identified as bisexual. While the Canadian Community Health survey captures a different age range than that served by Foundry, and self‐identified non‐heterosexual orientation may be more common in young people than older adults, this appears to be an over representation. Self‐reported sexual orientation in the Foundry user group included 14% bisexual, 4% gay or lesbian, 3% pansexual and 1% questioning. Seven percent identified as ‘other’ from listed categories.

#### 3.1.4 Housing status

One in 10 youth (10%) reported living in insecure housing situations and 13% reported not having any income source. The vast majority of youth (91%) reported parents or other family members as their guardian. Notably, 7% reported that their guardian was a social worker, service agency or other. Only 1% of youth reported living with foster parents. Youth aged 18–21 were more likely to report having insecure housing. Youth who identified as male, transgender or other were more likely to report having insecure housing than females.

### 3.2 Service utilization

**FIGURE 2:**
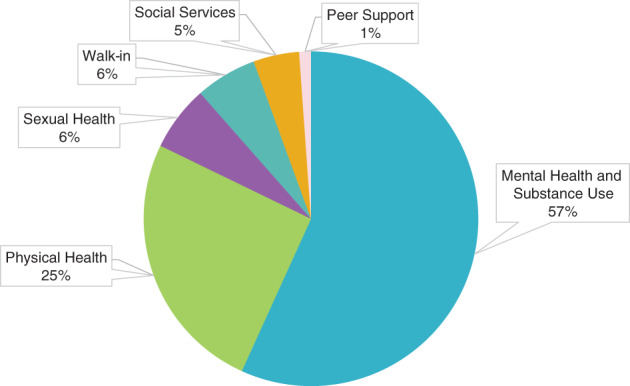
Distribution of services accessed during the proof of concept phase

**FIGURE 3:**
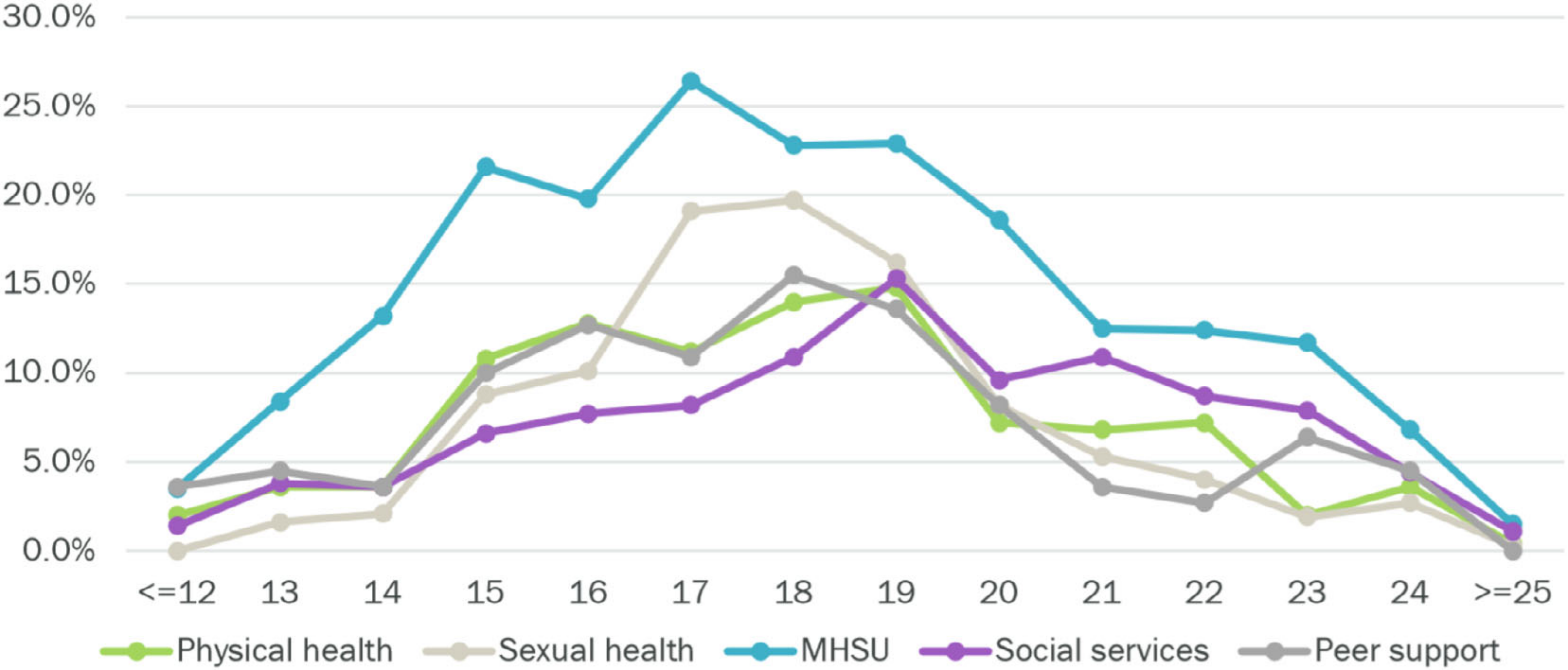
Services accessed by age sites included: Four out of six proof of concept sites (Prince George, Campbell River, Kelowna, and North Shore, excluding Vancouver and Abbotsford)

As shown in Figure 2, our data highlighted that, in a single visit, many youth access one or more of Foundry's five distinct services (i.e., primary care, mental health care, substance use support, peer support, and/or social services). The most common services requested and accessed across Foundry centres were MHSU services, accessed a total of 21 960 times, or 57% of all services. As shown in Figures 2 and 3, in the same period, primary care services (physical health and sexual health) were requested and accessed 9000 times, or one in four (25%) services. Youth aged 17 and 18 years accessed sexual health services at a relatively higher rate than younger and older peers. Youth aged 21 years and older accessed social services nearly as much as they accessed MHSU services (Figure 2).

### 3.3 Service referrals

Given the early focus on co‐design, Foundry sought to measure referral patterns, hinting at acceptability as well as an understanding of what youth viewed as help‐seeking alternatives to Foundry. Data showed that the most common referral sources for young people were there peers and their family members (45%), that is, lay people, signalling a significant and early credibility with the target population. Other referral sources were from family physicians (14%), school counsellors (13%), and the internet (12%).

### 3.4 Alternatives to foundry

As a way of exploring unmet need and early intervention, the question ‘where would you have gone today if not Foundry’ showed that nearly half (44%) responded ‘nowhere/I wouldn't have gotten help’. Another 22% reported that they would have turned to their family or friends (i.e., not accessed a professional helping service) for assistance. Additionally, the majority of youth (58%) who accessed Foundry during the proof of concept phase reported that they had not accessed a mental health or substance use service in the past year. This outcome is highly suggestive of acceptability and addressing unmet need early.

## 4 DISCUSSION

Within the proof of concept period, Foundry centres have been successful in reaching the target age group of youth between the ages of 12 and 24 primarily seeking support for MHSU use concerns. It is encouraging that the highest levels of service utilization are from youth ages 15–19, the age of onset for many mental health issues (Patel et al., 2015). It is also encouraging that Foundry centres appear to be serving a relatively large portion of youth marginalized by mainstream services, with vulnerabilities for mental or physical health issues, such as LGBTQ2+ orientation, those self‐identifying as non‐White or those having insecure housing. Based on the early results of this Proof of Concept phase, funding from the provincial government for an additional five (Phase 2: Victoria, Penticton, Ridge Meadows, Richmond, Terrace), eight (Phase 3: Surrey, Squamish, Langley, Kimberley‐Cranbrook, Burns Lake, Williams Lake, Comox, and Port Hardry) and four (TBD) centres were announced in 2017, 2020, and 2021 respectively. Moving forward, it is important that lessons learned are incorporated.

One key lesson was the variation in service utilization pattern by age (e.g., sexual health by female teens, social services by older youth). This variation highlighted the importance of having an integrated one‐stop health and social service delivery tailored to address the varying needs of young people across the 12–24 age range (Barbic et al., 2019; Halsall et al., 2019; Mei et al., 2020). With a significant majority of Foundry centres clients identified as female, the data suggest a need to continue efforts to normalize help seeking behaviours for males, especially those between the ages of 15 and 19 years (Zenone et al., 2020) and youth that identify outside the gender binary. The data also suggest a need to explore the needs of young youth (12–15), as these youth appear to be under‐explored in the literature in both Canada and International jurisdictions (Alleyne, 2018; Mei et al., 2020; Rider et al., 2018).

Another learning from the data was to further explore how best to provide culturally sensitive services in a good way. Although individuals identifying as Indigenous make up just 5.4% of the general population in BC, 46% of Indigenous people are below the age of 24 (compared to 29% for the non‐Indigenous population) (Government of British Columbia, 2017; Lang et al., 2018). In Canada, there have been numerous examples of data sources related to the health of Indigenous peoples that have not effectively contributed to the health of First Nations, Métis, or Inuit Peoples (Smylie et al., 2006). As our team works with Indigenous partners and communities to develop best approaches to data governance and mobilization, we chose not to report exact values in this paper. However, we do have permission to report that Indigenous youth were over‐represented as service users at Foundry centres during the proof of concept phase. This aligns with many other Canadian studies that show high needs for youth‐centred services for Indigenous youth (Chandler, 2011; Kidd et al., 2019). Opportunity exists to engage in the co‐development of a culturally responsive model to support Indigenous youth accessing Foundry. This may include participatory consultation and engagement to work towards a culturally humble and safe model that grows to support the diverse needs of BC youth (NEJM Catalyst, 2017).

Our shared learnings also include an understanding that future youth and family engagement requires organizations to create opportunities for input on strategies to draw in those young people choosing not to enter Foundry centres. Although the collection of metrics such as service access, service type, and demographics is important, an aspirational outcome of the Foundry initiative is to show transformation to access, notably by creating increased availability in part through service co‐location and integration and the development of low barrier services such as peer support and solution‐focused brief therapy, well suited for walk‐in counselling. Improved access or system integration were not measured here and future work with young people and communities is needed to co‐develop ways to capture effective integration and community engagement efforts.

Finally, our findings contribute to an emerging body of literature on integrated youth health services (Hetrick et al., 2017; McGorry et al., 2019; Mei et al., 2020; Rickwood et al., 2019; Settipani et al., 2019). The first fully operating Foundry centres show promising results about improving young people's experience of care and achieving positive outcomes. Based on the distribution of service use, early evidence exists for Foundry centres building reliable places for young people to access health and social services. With over half of new Foundry clients reported that they were referred to the centre by a friend, family member, or school counsellor/teacher, the data also showed a promising source of referrals that were outside of the traditional medical model. Our results also suggest that Foundry is meeting an unmet need in these pilot communities (with most youth reporting they would have gone nowhere for help). While significant progress has been made to understand the illness and self‐management needs of youth with mental illness, little attention has been focused on co‐designing accessible spaces to meet their needs. The evidence base for understanding the impact of integrated youth health services on community integration and urban design is, to date, underdeveloped. Future work is needed to engage diverse young people across the province to ensure ongoing momentum from Phase 1, and to share learnings to optimize how young people can learn about Foundry and access services where and when they need them.

### 4.1 Limitations

Our team learned that collecting data across a network of centres with individual electronic medical records within unique health authorities posed challenges. A customized electronic data collection platform, with strong data quality assurance and data governance measures, is needed to support consistent and meaningful data collection, providing a common registry for all youth and caregivers interacting with Foundry centres and allowing for the collection of a common evaluation and quality improvement data set and network practice guidelines that can be rolled out across the network of services. Other limitations include the mixed methodology used to collect the data. Given the absence of a common data collection tool, data were collected from several sources including paper forms and electronic medical record systems. Additionally, data fields for service utilization were not uniform for the centres. For instance, at one centre, substance use visits were coded as mental health visits, leading to under‐representation of one and over‐representation of another. Access data is also reflective of the supply of a given service rather than the demand. For instance, initial funding constraints have limited primary care availability, which is reflected in the service utilization data. As Foundry's service model matures, ongoing systematic data collection, including regular measures of youth outcomes and experience of care, will support the centres to improve service provisions in areas where youth report lower levels of satisfaction. Routine use of youth feedback will also ensure that Foundry maximizes the engagement of young people in having a say in their own care, a key component to Foundry's overall approach to care and consistent with its guiding principles. To avoid a strong selection bias of youth already using the centre, sampling young people who opted not to return or never to access a centre, would be more helpful to inform access.

## 5 CONCLUSION

This study provides early evidence that Foundry is establishing itself as a branded network of youth‐friendly, inviting, accessible centres that are bringing together numerous partners to deliver a wide range of services to meet the needs of young people and their caregivers. The investment by government, philanthropists and stakeholders has led to a reduction in accessibility barriers to an array of services supporting the health and wellness of young people and their families.

## CONFLICT OF INTEREST

The authors declare no conflicts of interest.

## Data Availability

The data that support the findings of this study are available on request from the corresponding author. The data are not publicly available due to privacy or ethical restrictions.
